# Exploration of Methods for In Situ Scale Removal During Magnesium Hydroxide Membrane Crystallization

**DOI:** 10.3390/membranes15090267

**Published:** 2025-09-03

**Authors:** Ester Komačková, Lukáš Sedlák, Ivan Červeňanský, Jozef Markoš

**Affiliations:** Institute of Chemical and Environmental Engineering, Faculty of Chemical and Food Technology, Slovak University of Technology, Radlinského 9, 812 37 Bratislava, Slovakia; ester.komackova@stuba.sk (E.K.); lukas.sedlak@stuba.sk (L.S.); jozef.markos@stuba.sk (J.M.)

**Keywords:** membrane crystallization, magnesium hydroxide, hollow fiber anion-exchange membrane

## Abstract

In coastal countries facing a shortage of drinking water, seawater desalination is essential for the production of potable water. During desalination, a large volume of waste stream, known as brine, is generated. This stream contains high concentrations of salts, particularly those of economic importance to the European Union, such as magnesium and calcium. By further processing this stream, these materials can be recovered. One method studied for separating magnesium from wastewater is membrane crystallization (MCr). The MCr process developed in this work utilizes ion-exchange membranes that separate the model brine solution from a precipitating agent, which is a solution of sodium hydroxide. During the process, the membrane allows the transport of anions between the two solutions, enabling the reaction between OH^−^ anions and Mg^2+^ cations, which leads to the formation of a magnesium hydroxide precipitate. The formed precipitate can then be filtered out of the brine solution, which now has decreased salinity due to crystallization facilitated by the ion-exchange membrane. However, precipitation occurs near the membrane surface, resulting in the deposition of magnesium hydroxide onto the outer surface of the membrane. The aim of this study is to investigate methods for effectively removing magnesium hydroxide from the membrane surface, with a primary focus on maximizing the yield of magnesium hydroxide crystals in suspension. Crystal removal was induced by circulation of hydrochloric acid, followed by circulation of demineralized water through the membrane module after crystallization. In this study, a membrane module made of hollow-fiber anion-exchange membranes was employed. The production cost of these membranes is approximately 50% lower per square meter compared to flat-sheet membranes commonly used in electrodialysis, demonstrating strong potential for commercial application. More than 85% magnesium conversion was achieved during the process, yet the majority of the crystals remained attached to the membrane. Circulation of hydrochloric acid and demineralized water after the crystallization process caused detachment of the crystals into suspension, nearly doubling their yield.

## 1. Introduction

The global human population continues to grow, and projections indicate that this trend will persist [1]. With the rising number of inhabitants, demands for a higher standard of living are also increasing. In many coastal regions, people have begun constructing desalination plants to process seawater for the production of clean drinking water. These facilities primarily utilize membrane technologies, especially reverse osmosis (RO) [2]. However, processing seawater through RO generates a waste stream with a high concentration of dissolved salts, several times higher than in the original seawater [3,4]. Table 1 shows a comparison of cation concentrations between sea water and different types of exhausted brines.

Approximately 140 million m^3^ of brine are produced globally each day by reverse osmosis or mining [7]. Common methods for brine disposal include discharging into the ocean [8], surface water [9], and sewage system [10], which poses significant environmental problems. These methods often involve high costs and environmental risks, underscoring the need for more sustainable and cost-effective solutions [11].

The European Union annually determines a list of critical raw materials that are in high demand on the European market and carry a high risk of supply disruption to the EU. Among these are substances found in brines [12]. One such valuable material is magnesium, [13,14]. Magnesium, in the form of magnesium hydroxide, has a wide range of industrial applications. It can be used not only as a flame-retardant [15,16,17] but also as an antibacterial agent [18] or for reducing water pollution caused by heavy metals [19,20,21].

Research into the reactive crystallization of magnesium hydroxide from magnesium chloride solution with sodium hydroxide as a precipitating agent began almost 50 years ago [22,23]. These early experiments showed that direct contact between magnesium chloride solution and precipitating agent produces very fine particles, which tend to agglomerate, forming a gelatinous suspension that is difficult to separate. In subsequent years, studies explored the effects of operational conditions on reactive crystallization to achieve the smallest possible agglomerates [5,24,25].

Cipollina et al. [4] proposed a desalination process concept for exhausted brine from saltworks in Trapani, Italy, which included the precipitation of magnesium in the form of Mg(OH)_2_. The authors then continued their research using a semi-batch crystallizer and a continuous crystallizer [26]. However, purity of produced Mg(OH)_2_ crystals is significantly impacted by co-precipitation. The precipitation of Mg(OH)_2_ may be accompanied by the precipitation of calcium in the form of Ca(OH)_2_, occurring at a higher pH within the same process. Vassallo et al. [6] tested a pilot plant for the crystallization of magnesium and calcium. But in reactive crystallization, the direct contact between the processed brine and the precipitating agent can cause unintended issues in the precipitation process, such as co-precipitation, of calcium in the form of Ca(OH)_2_, occurring at a higher pH within the same process which affects the product’s quality [27].

**Figure 1:**
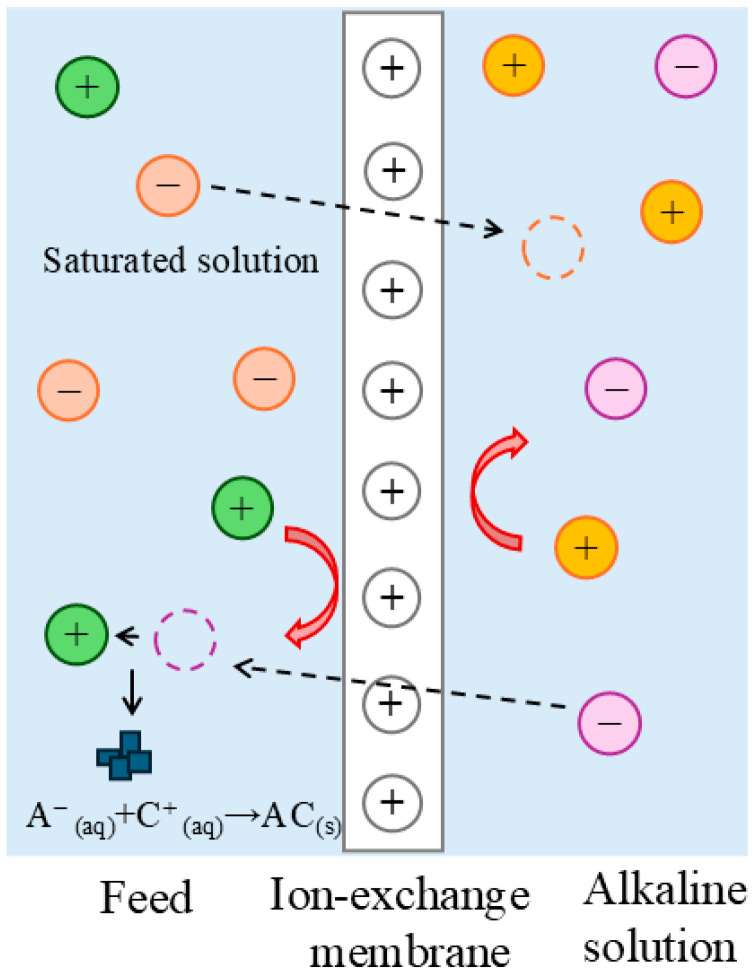
Reactive membrane crystallization using ion-exchange membrane.

Membrane technologies, such as electrodialysis using ion-exchange membranes, are also employed for the valorization of raw materials from waste brines. This led to the idea of combining membrane technology with the precipitation crystallization process for magnesium recovery, which has been patented by La Corte et al. [27] as an ion-exchange membrane crystallizer. This ion-exchange membrane crystallizer consisted of a flat-sheet anion-exchange membrane that separated the brine from the alkaline reactant which flowed through the serpentine channel on opposite sides of the membrane. The basic principle of the process operation is depicted in Figure 1. The anion-exchange membrane selectively allowed only anions to pass through, causing hydroxide anions to migrate to the side of the brine rich in magnesium cations. Supersaturation of the solution with OH^−^ ions was achieved rapidly, and at a pH of approximately 9.8 [6,28] an immediate reaction occurred between the hydroxide ions according to the following reaction:

(1) $${\mathrm{Mg}}^{2+}\left(\mathrm{aq}\right){+2\mathrm{OH}}^{-}\left(\mathrm{aq}\right)\to \mathrm{Mg}{\left(\mathrm{OH}\right)}_{2}\left(s\right)$$

At the same time, chloride anions from the brine migrated to the alkaline reactant side to maintain electroneutrality. In general, it is assumed that ions with the same charge as the membrane were repelled based on the Donnan exclusion theory [29,30]. Ion transport across the membrane occurs not only due to the concentration gradient within it, but also due to the electrochemical potential gradient, which arises from the maintenance of Donnan equilibrium at the membrane-solution interface [28].

One of the significant advantages of membrane crystallization is that there is no direct contact between the alkaline solution and the brine, thus preventing the co-precipitation of other substances that could contaminate the product and affect its final quality. The second advantage is decreasing brine conductivity and salinity due to the crystallization of magnesium ions. In contrast, the direct addition of a precipitating agent to the brine also introduces sodium ions. Furthermore, the integration of membrane crystallization with electrodialysis using bipolar membranes could enhance the sustainability of the process by enabling the regeneration of the precipitating agent while simultaneously producing hydrochloric acid, which is also required for the proposed membrane crystallization process. Although studies report achieving high magnesium recovery efficiency, the issue of membrane fouling is only marginally mentioned. Upon reaching supersaturation of the solution with OH- ions, most magnesium cations react with hydroxide anions near the membrane surface, where the supersaturation is highest. Consequently, most of the product may remain trapped on the membrane rather than forming in the solution. La Corte et al. [27] achieved with their membrane crystallizer high magnesium recovery efficiency and high product purity, while also reporting that a thin layer of magnesium hydroxide was attached to the membrane. They concluded that the formed layer did not affect the subsequent experiments, which were performed without previous cleaning of the membrane. Vassallo et al. [28] also mentioned the issue of membrane fouling but mitigated it by positioning the membrane module horizontally and adding cleaning balls, which were circulating in the brine channel, whose purpose was to hinder the formation of scaling during the experimental run.

Membrane fouling has been extensively investigated, primarily in the context of membrane crystallization using membrane distillation, whereas reactive membrane crystallization remains largely unexplored in this regard. Fouling mitigation strategies employed in membrane crystallization with membrane distillation include pre-treatment, membrane bubbling, flushing, backflow application, chemical cleaning, and membrane modification [31]. The application of pre-treatment methods depends on the brine composition and works on the principle of reducing the concentration of species that cause fouling. These methods include processes such as low-pressure membrane filtration, coagulation and flocculation, adsorption, pH adjustment, and the addition of anti-scalants. Gas bubbling can mitigate external fouling by increasing the shear rate and enhancing fluid dynamics on the membrane surface [32]. Membrane flushing commonly uses deionized water to remove solutes from the membrane surface [31]. The application of backflow reverses the driving force of the process, thereby reducing surface interactions between the membrane and scalants [33]. Chemical cleaning is commonly employed to disrupt foulant–membrane interactions [32]. Membrane distillation crystallization relies on strongly hydrophobic membranes that inherently resist fouling; thus, surface modification offers an effective strategy to further minimize scaling. An interesting approach is the development of self-cleaning membranes capable of sustaining stable separation for multiple cycles [34]. Since reactive membrane crystallization with ion-exchange membranes operates differently from membrane distillation, fouling mitigation should primarily rely on removing scale deposits from the external surface, as ions of the same charge are repelled by the membrane and thus the formation of crystals inside the membrane is unlikely. An alternative approach could be to prevent the formation of scales in the first place, through improved control of nucleation, which could be significantly altered by changing the volume-to-surface ratio, as shown by Odua et al. [35] for membrane distillation crystallization.

Previous studies have confirmed that magnesium can be separated in the form of magnesium hydroxide crystals with high purity and high recovery efficiency, or with high conversion rates, through reactive membrane crystallization. They also reported that crystals form on the other surface of the membrane but do not significantly impede the anion-exchange process. However, these studies do not report the percentage of crystals present in the suspension and therefore do not provide insight into the extent of membrane fouling that may occur during the process. In this work, an experimental hollow fiber anion exchange membrane is applied within the reactive membrane crystallization setup. The main objective is not only to recover magnesium hydroxide from model solution but also to investigate in situ cleaning methods for removing crystals from the membrane surface, thereby increasing their yield in suspension. These methods incorporate chemical cleaning with hydrochloric acid solution and demineralized water, which was delivered directly inside the hollow fiber in a backflow configuration, meaning that the protons needed to diffuse from the internal membrane surface through the anion-exchange membrane itself to the external membrane surface, where partial dissolution of deposited Mg(OH)_2_ occurs.

## 2. Materials and Methods

### 2.1. Materials

In this work, the solution of magnesium chloride was used as a model solution of brine. Magnesium chloride hexahydrate MgCl_2_·6H_2_O (technical grade, purity > 98%), NaOH pellets (technical grade) and 6 M solution of HCl were all supplied by CentralChem, Slovakia. The experimental apparatus was built from Eppendorf (Eppendorf SE, Hamburg, Germany) BioFlo^®^ 320 control unit, multimeter Thermo Scientic (Waltham, MA, USA) PC Eutech 450 for pH measurement, laboratory scale KERN PCB 3000-2, IKA MICROSTAR 15 control equipped with Rushton turbine for solution mixing.

**Figure 2:**
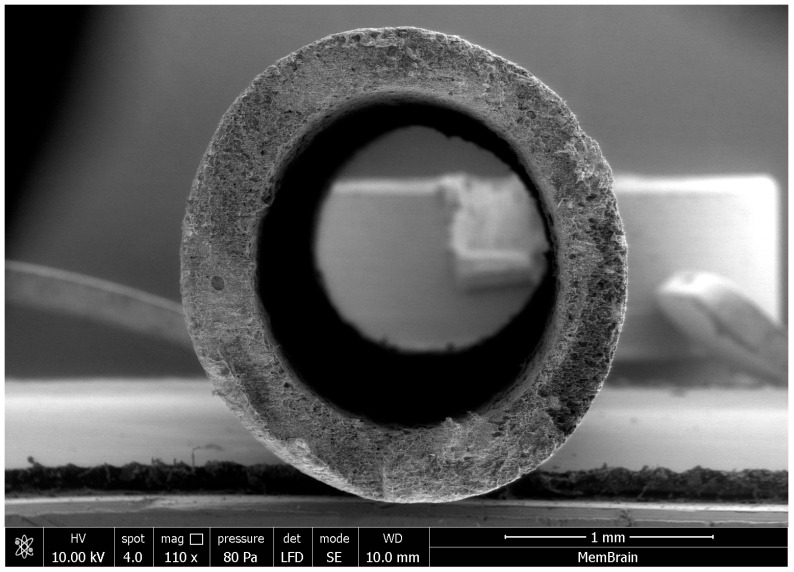
SEM image showing the cross-section of the ion-exchange hollow fiber manufactured by MemBrain s.r.o.

Experimental, commercially unavailable anion-exchange membranes in the form of hollow fibers, developed by MemBrain s.r.o. (Stráž pod Ralskem, Czech Republic), were used in this study. Their fabrication is similar to that of flat-sheet RALEX^®^ membranes: the ion-exchange resin is ground, mixed with polyethylene, homogenized, and melted to prepare a granulate consisting of a uniform mixture of both components. This granulate is then fed to the extrusion head in a melt-spinning process, producing hollow fiber membranes generally regarded as dense. A scanning electron microscope (SEM) image of the cross-section of the experimental anion-exchange hollow fiber membrane is shown in Figure 2.

These membranes present a promising option for large-scale commercial implementation, as their cost per square meter is only half that of flat-sheet membranes produced by MemBrain s.r.o. The membrane module consists of a single hollow fiber membrane with a length of 1.5 m. The properties of the membrane are shown in Table 2.

**Figure 3:**
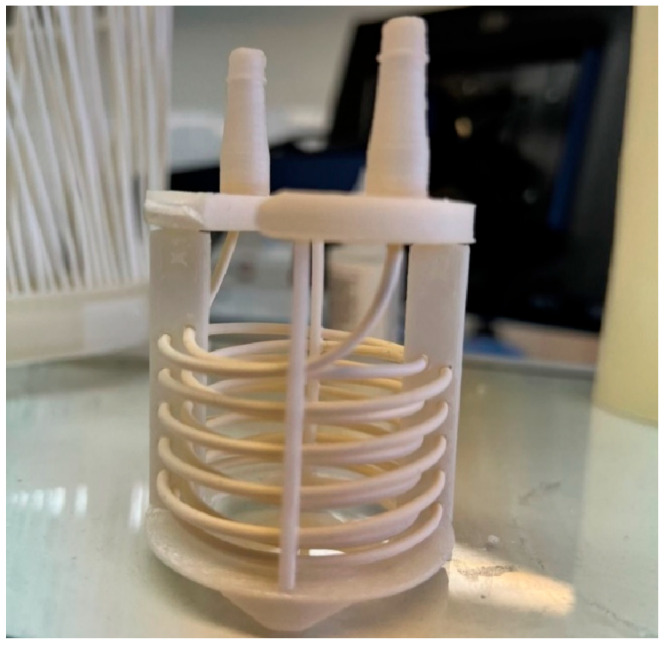
A single hollow fiber anion-exchange membrane module.

All other components of the membrane module were fabricated from polypropylene using a BambuLab 3D printer. The membrane module used in presented experiments is shown in Figure 3 and it consisted of a single hollow fiber wound around custom 3D-printed supports.

### 2.2. Setup Description

Before use, the membrane module was soaked in water for one hour to allow the membrane to swell, followed by activation. Activation of the membrane module consisted of submerged into the 0.5 mol/L HCl for one hour.

**Figure 4:**
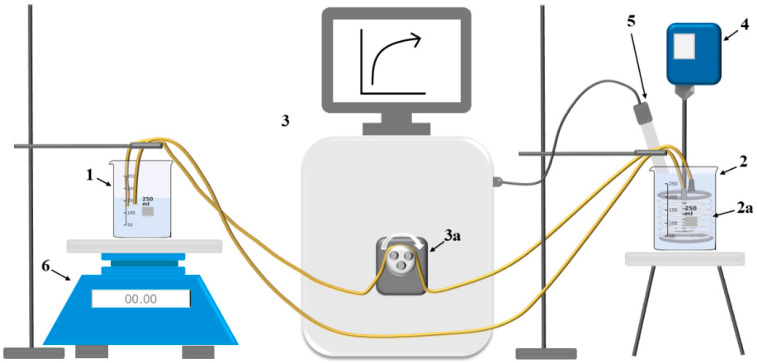
Sketch of the experimental apparatus, 1—reservoir with alkaline solution, 2—reactor with model brine solution, 2a—hollow fiber membrane module, 3—Eppendorf BioFlo^®^ 320 control unit, 3a—peristaltic pump, 4—mixer, 5—pH probe, 6—scales.

Magnesium hydroxide membrane crystallization was studied using the apparatus depicted in the simplified scheme in Figure 4. The model brine solution located in reactor (No. 2) in which the membrane module was submerged (No. 2a) was continuously stirred throughout the process by overhead stirrer (No. 4). This improved the transfer of OH^−^ ions across the laminar layer on the brine side, leading to the reaction between OH^−^ ions and Mg^2+^ ions according to Equation (1).

The pH of the model brine solution in the reactor was continuously monitored during the process by pH probe (No. 4) as the crystallization of Mg(OH)_2_ is highly sensitive to pH, occurring within the pH range of 9.8 to approximately 10.4 [28]. After reaching the pH value of 10.4, it was assumed that complete conversion of Mg^2+^ ions occurred [36].

On the other hand, if pH values were higher than 10.4, it could lead to co-precipitation of other components found in real brine, e.g., co-precipitation of Ca^2+^ in the form of Ca(OH)_2_. Co-precipitation of other components or ion sorption into the crystal structure could degrade the quality of the product [28].

The weight of the alkaline solution (No. 1) was periodically recorded using scales, No. 6, to verify the functionality of the module and ensure that no damage occurred, which would result in direct contact between the model brine solution and the alkaline solution. If the module was damaged, allowing direct contact between the two solutions, this would be reflected in a significant rise in pH values. Otherwise, the weight of the reservoir changed minimally, within a range of 5 g. These small changes could be attributed either to evaporation of the alkaline solution or the formation of bubbles in the silicone tubes.

### 2.3. Experimental Procedure

All experiments were conducted with a model solution, consisting only of dissolved MgCl_2_ and representing the brine, which was placed in the reactor containing the hollow fiber anion-exchange membrane module. The alkaline NaOH solution, serving as the precipitating agent, was held in the reservoir. The 125% excess of OH- ions was selected based on our previous studies, in which a high excess of OH- ions ensured a more stable driving force for mass transfer in batch configuration. Experimental conditions are presented in Table 3.

The alkaline solution was pumped from the reservoir into the membrane module through silicone tubing using a peristaltic pump at a flow rate of 14 mL/min. The outgoing precipitating agent was recirculated back to the reservoir. Chosen initial concentrations of MgCl_2_ were selected based on the published work by Cipollina [26]. The reactor and the reservoir were mechanically stirred at 600 rpm and 100 rpm, respectively. The pH values and flow rates were recorded and controlled using an Eppendorf BioFlo^®^ 320 control unit. Additionally, the mass inside the reservoir was monitored. The duration of each experiment was 5 h.

The regeneration process of the membrane module after each experiment involved submerging the membrane module in 125 mL 0.5 mol/L HCl and circulating it through the system for one hour, followed by multiple rinsing procedures with 125 mL demineralized water. The demineralized water in the rector was exchanged until the conductivity of demineralized water dropped below 1 mS/cm.

### 2.4. Methodology

The entire process was divided into three stages. The first stage consisted of the membrane crystallization of Mg(OH)_2_. The process parameters for the first stage remained unchanged throughout all experiments and the parameters are presented in Table 2. The following two stages were introduced in order to study their application for removal of crystals from the membrane surface. The second stage involved the circulation of HCl through the membrane module and the third stage involved the circulation of demineralized water through the membrane module. Variations in the process parameters were made in the second and third stage in order to examine the effect of these process parameters on the crystal yield in the suspension. Throughout all three stages the suspension and the membrane module remained in the reactor. Process parameter changes were made only in one stage in individual experiments in order to evaluate their effects.

Circulation of the concentrated HCl through the hollow fiber causes the repulsive forces of the anion-exchange groups in the membrane to be insufficient to repel cations, which results in the membrane starting to lose its selectivity, therefore allowing these co-ions to pass through the membrane [30]. The introduction of HCl was intended to remove a thin layer of the crystals closest to the membrane surface, according to Equation (2), allowing the upper layer of crystals to be released into the suspension. This would increase the crystal yield in the suspension and simultaneously generate potential crystal nuclei in the suspension that could continue to grow.

(2) $$\mathrm{Mg}{\left(\mathrm{OH}\right)}_{2}\left(s\right)+2\mathrm{HCl}\left(\mathrm{aq}\right)\to {\mathrm{MgCl}}_{2}\left(\mathrm{aq}\right){+2H}_{2}O\left(l\right)$$

The third stage involved the circulation of demineralized water in the membrane module, which aimed to dislodge crystals in the vicinity of the membrane surface into the suspension. Demineralized water was pumped through the fiber placed in the reactor and due to osmosis and hydrodynamic pressure inside the fiber water was transported through the membrane to the reactor.

#### 2.4.1. Modification in the First Stage

**Figure 5:**
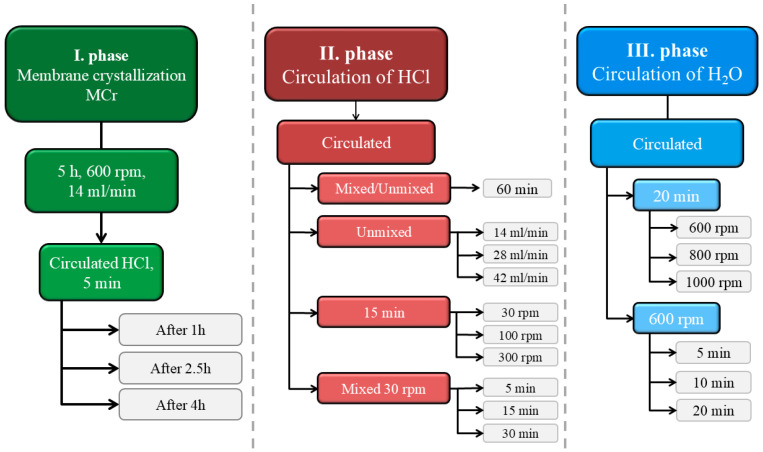
Overview of the experiments performed with the modifications examined at each stage.

As mentioned above, the process parameters of the first stage which were presented in Table 2 were not altered. However, in this set of experiments the effect of 2 mol/l HCl circulation on the crystal yield in the suspension of HCl was investigated if the acid was introduced for a short period of time during the first stage. The introduction of HCl during the first stage occurred at different times from the beginning of the experiment—after 1 h, after 2.5 h, or after 4 h, see Figure 5.

#### 2.4.2. Modifications in the Second Stage

In the second stage, four sets of experiments were conducted, see Figure 5. In the first set of experiments, two extremes were examined: stirring the reactor at 600 rpm or not stirring the reactor at all, both for a duration of 60 min. In the second set of experiments, the effect of varying the volumetric flow rate of HCl on the crystal yield in the suspension was investigated. In this set, the suspension was not stirred, and HCl was circulated in the system for 15 min, with the volumetric flow rate of HCl increasing from 14 mL/min to 28 mL/min and then to 42 mL/min. In the third set of experiments, HCl was circulated in the system for 15 min, and the effect of changing the suspension stirring speed inside the reactor on the crystal yield was examined. The stirring speed varied from 30 rpm to 100 rpm, and then to 350 rpm. In the final set of experiments, the suspension was stirred at the lowest possible stirring frequency, 30 rpm, and the effect of varying the circulation time in the system was investigated, with HCl circulating for either 5 min, 15 min, or 30 min. At these low mixing speeds, significant local concentration gradients could have occurred, which might have affected the results after the second stage. The actual outcome of the second stage will only be confirmed in the third stage, which is why the third stage is also displayed in the final graphs.

#### 2.4.3. Modifications in the Third Stage

In the third stage, the effects of the circulation time of demineralized water in the membrane module and the intensity of suspension stirring on the crystal yield in the suspension were investigated, see Figure 5. In the first set of experiments, demineralized water circulated in the system for 20 min, with three tests conducted at different stirring speeds of 600 rpm, 800 rpm, and 1000 rpm. In the second set of experiments, the suspension was stirred at 600 rpm, while demineralized water circulated in the system for either 5 min, 10 min, or 20 min.

### 2.5. Data Collection and Sampling Procedure

To determine the efficiency of membrane crystallization, it was necessary not only to collect samples but also to gather and record data. The collected data included pH values, which were automatically recorded by the Eppendorf BioFlo^®^ 320 control unit and additionally logged at each sampling point. During the first half of the first stage, samples were collected more frequently—every 30 min. In the second half of the first stage, samples were taken every hour. During the second and third stages, samples were again collected more frequently, every 5 min.

The samples were diluted to fall within the calibration range. Two types of samples were taken every hour. Type I was used for determination of the free dissociated Mg^2+^ ions. This sample was first filtered through a 0.45 µm syringe filter and then diluted with demineralized water. Type II was used for the determination of all Mg^2+^ ions present in the suspension. This sample was diluted with 0.5 mol/L HCl, whose role was to dissolve any formed crystals of Mg(OH)_2_ and thus convert the magnesium back to the dissociated form.

From the analysis of these two sample types, we were able to determine the concentration of crystals in the suspension according to Equation (3).

(3) $$C_{Mg{\left(OH\right)}_{2},suspension}=C_{Mg^{2+},total}-C_{Mg^{2+},\left(aq\right)}$$

$C_{Mg{\left(OH\right)}_{2},suspension}$ represents the concentration of magnesium hydroxide in the suspension, $C_{Mg^{2+},total}$ is the total concentration of Mg^2+^ ions present in the suspension at a given sample time (sample type II), and $C_{Mg^{2+},\left(aq\right)}$ refers to the concentration of free Mg^2+^ ions at a given sample time (sample type I).

The concentration of all Mg(OH)_2_ crystals (in the suspension and on the membrane) $C_{Mg{\left(OH\right)}_{2},total}$ was calculated as the difference between the initial concentration of Mg^2+^ ions in the model solution $C_{Mg^{2+}}^{0}$ and the concentration of free Mg^2+^ ions in the suspension (sample type I) according to Equation (4).

(4) $$C_{Mg{\left(OH\right)}_{2},total}=C_{Mg^{2+}}^{0}-C_{Mg^{2+},(aq)}$$

The recovery of Mg(OH)_2_ crystals in the suspension $Y\left(Mg{\left(OH\right)}_{2,suspension}\right)$ was calculated as the difference between the concentration of all Mg^2+^ ions present in the suspension (sample type II) and the concentration of free Mg^2+^ ions (sample type I), divided by the initial concentration of Mg^2+^ ions in the model brine solution as defined by Equation (5). Similarly, the percentage of Mg(OH)_2_ crystals on the membrane $Y\left(Mg{\left(OH\right)}_{2,membrane}\right)$ can be determined with the known concentration of Mg(OH)_2_ crystals in the suspension using Equation (6). Overall conversion to Mg(OH)_2_, referred to as $Y\left(Mg{\left(OH\right)}_{2}\right)$, was calculated according to Equation (7), using the initial concentration and the concentration of free Mg^2+^ ions (sample type I).

(5) $$Y\left(Mg{\left(OH\right)}_{2,suspension}\right)=\frac{C_{Mg^{2+},total}-C_{Mg^{2+},(aq)}}{C_{Mg^{2+}}^{0}}\times 100\%$$

(6) $$Y\left(Mg{\left(OH\right)}_{2,membrane}\right)=\frac{C_{Mg{\left(OH\right)}_{2},total}-C_{Mg{\left(OH\right)}_{2},suspension}}{C_{Mg^{2+}}^{0}}\times 100\%$$

(7) $$Y\left(Mg{\left(OH\right)}_{2}\right)=\frac{C_{Mg^{2+}}^{0}-C_{Mg^{2+},(aq)}}{C_{Mg^{2+}}^{0}}\times 100\%$$

Aqueous samples containing dissociated magnesium were analyzed using an Agilent Infinity 1260 system (Agilent Technologies, Santa Clara, CA, USA) equipped with a cation-exchange column Hamilton PRP-X-800, 4.1 mm × 250 mm, 7 µm, and each sample was analyzed in triplicate. HPLC was employed for indirect absorbance detection at 220 nm with a reference wavelength of 360 nm. The mobile phase consisted of a 1.5 mmol/L CuSO_4_ aqueous solution with 0.3 mmol/l H_2_SO_4_, and volumetric flow of mobile phase was 1 mL/min.

## 3. Results and Discussion

In each experiment, more than 85% magnesium conversion was achieved during the first stage. On average, after the crystallization stage, only 1% of Mg(OH)_2_ crystals were found in suspension, while the remainder were present on the membrane surface. The results shown below represent the percentages of dissociated magnesium cations, crystals in suspension, and crystals on the membrane, relative to the initial amount of Mg^2+^ ions calculated according to Equations (5) and (6), and are presented as bar charts. To facilitate interpretation, mostly the results from the third (final) stage of one experimental set are compared.

### 3.1. Evaluation of Single Experiment

**Figure 6:**
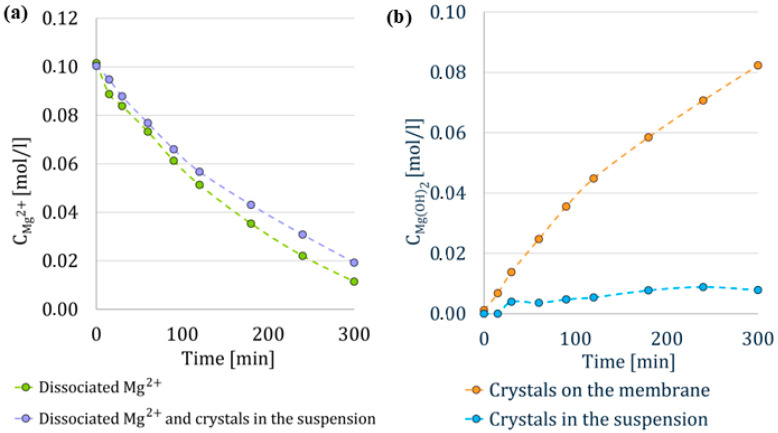
Concentration profiles during the experiment: (**a**) change of Mg^2+^ ions concentration in the reactor through the MCr; (**b**) concentration of Mg(OH)_2_ in the reactor through all three stages of the experiment.

The crystallization of magnesium hydroxide depends on the supersaturation of OH^−^ ions in the reactor, which diffuse through the membrane from the precipitating agent. Once supersaturation is achieved crystals begin to form spontaneously. The concentrations of dissociated Mg^2+^ ions and crystals in the suspension were determined from the samples. Analysis of the samples revealed that the concentration of dissociated magnesium cations decreased during the MCr process, see Figure 6a, which corresponded to the formation of crystals in the solution and on the membrane, see Figure 6b. After the first stage, the concentration of dissociated Mg^2+^ ions was approximately 10 mmol/L, indicating that the remaining Mg^2+^ ions had reacted to form magnesium hydroxide. Figure 6a illustrates the trend in the consumption of dissociated magnesium cations and the simultaneous production of crystals in the solution. The difference between these trends is minimal, already showing that only a small portion of crystals were present in the suspension. Figure 6b directly plots the concentration of crystals in the suspension and it can be seen that their concentration was not excessively high, and most of the crystals remained retained on the membrane, where the supersaturation of OH^−^ ions in the solution was the highest. Based on these results we can estimate the flux of anions through the membrane assuming that the precipitation is instantaneous and the production of Mg(OH)_2_ is limited by mass transfer of OH^−^ ions through the anion-exchange membrane. The average flux of ions through the membrane was 5.58 × 10^−5^ mol/m^2^/s, which is an order of magnitude lower than the flux reported by Vassallo et al. [28], who used flat sheet membranes in their experiments that were thinner than the hollow fiber membranes used in our work.

**Figure 7:**
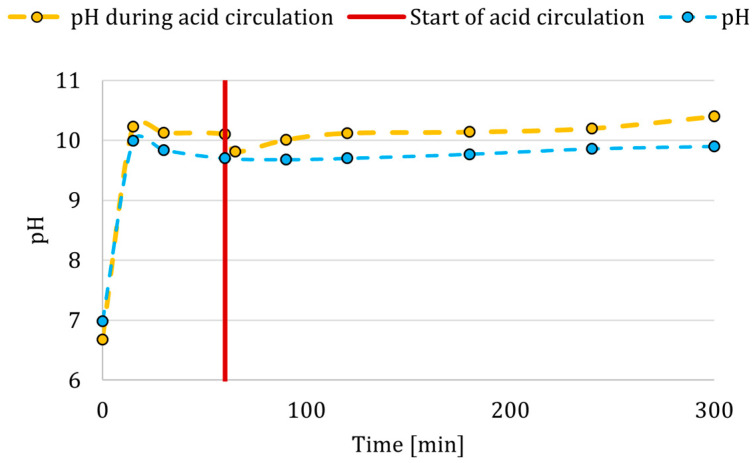
pH profiles in the model experiment and during the first stage with the HCl introduction to the fiber after one hour (red vertical line).

The production of Mg(OH)_2_ is also evident from the pH measurements in a typical membrane crystallization experiment conducted using the setup shown in Figure 7, as indicated by the blue line. The diffusion of OH^−^ ions through the anion-exchange membrane is indicated by an initial sharp increase in pH. This is followed by a gradual decrease as supersaturation is reduced due to Mg(OH)_2_ formation. Subsequently, the pH rises again as Mg^2+^ ions are progressively consumed, requiring slightly higher OH^−^ concentrations to sustain further precipitation. To maintain crystallization, the OH^−^ ion concentration (and thus pH) must continue to rise in order to meet the solubility product requirement.

### 3.2. Circulation of HCl in the 1st Stage

**Figure 8:**
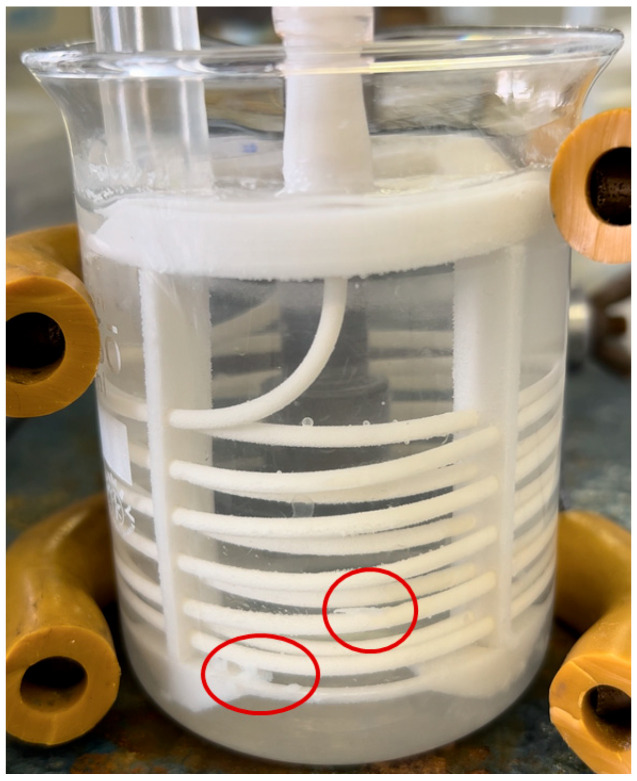
Photo of the experiment during the third stage showing large scales detached from the membrane.

The circulation of HCl in the system is described in more detail in the Section 2.4.1. During the introduction of HCl in the first half of the first stage, that is, after the first hour and after 2.5 h from the start of the experiment, the difference between the percentages of crystals in suspension was not as significant as the percentage difference after the introduction of HCl after the fourth hour of the experiment. It can be concluded that the circulation of HCl in the first half of the first stage had almost the same effect on the percentage of crystals in suspension because a sufficiently thick layer of crystals was not formed on the membrane to be disrupted by HCl and thus capable of detaching. Instead, a small amount of crystals on the membrane surface dissolved and reduced the supersaturation of the solution with OH^−^ ions in the reactor, which was reflected in the pH values, see orange line in Figure 7. Effectively removing part of the crystals from the membrane surface (Figure 8) would reduce the resistance to OH^−^ ions transfer, which could also be responsible for the overall higher conversions.

**Figure 9:**
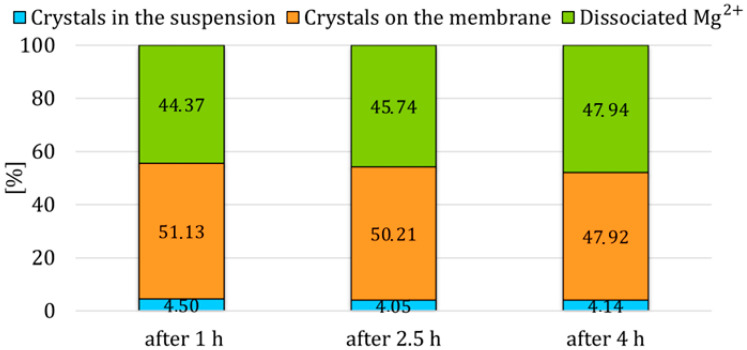
Percentage of Mg^2+^ ions in different forms at the end of the experiments. 1st column—introducing of HCl after the 1st hour; 2nd column—introducing of HCl after the 2.5; 3rd column—introducing of HCl after the 4th hours.

The greatest impact of HCl circulation in the first stage was observed when HCl was circulated in the system after the fourth hour, when the crystal yield was 9.29%. In this case, a sufficiently thick layer of crystals was formed on the membrane, and during the circulation of HCl, the lower layer of crystals on the membrane surface dissolved while the upper layer was released into the suspension. After four hours, a thicker layer of crystals formed on the membrane. Consequently, when HCl was circulated after four hours, a larger portion of the crystals, 9.29% detached into the suspension compared to circulation after one hour, 3.05%. Since a greater portion of the crystals was removed into the suspension, the resistance to the transfer of H^+^ ions during HCl supply in the second stage decreased. This led to the dissolution of the crystals, thereby reducing their yield after the third stage. On the other hand, if crystal nuclei had formed on the membrane within the first hour of the experiment, the introduction of HCl after one hour led to their dissolution. However, after completing all three stages of experiments, the final yield of magnesium hydroxide crystals in suspension after the third stage was nearly identical across all three experiments in this set, see Figure 9.

### 3.3. Evaluation of Modifications Applied in the 2nd Stage

The results are described in four chapters, with the graphs showing only the outcomes after the completion of the process. The graphs illustrate the percentage of reacted or dissociated magnesium cations, respectively.

#### 3.3.1. Impact of Mixing Suspension in the Reactor

**Figure 10:**
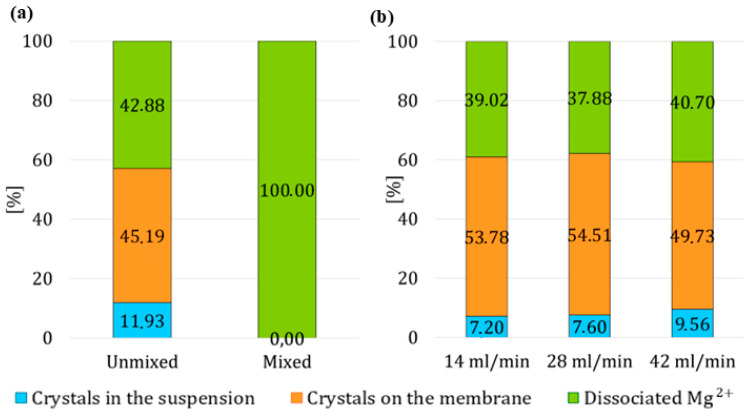
The percentage of reacted and dissociated Mg^2+^ ions after the third experimental stage: (**a**) impact of suspension mixing, (**b**) varying flow rate of acid.

High stirring speed of 600 rpm improved the mixing of the suspension within the reactor, potentially causing crystals to detach into the suspension. Additionally, vigorous stirring reduced the laminar boundary layer near the membrane surface on the suspension side, enhancing the transfer of H^+^ ions into the suspension. This increased ion transfer lowered the solution’s supersaturation, leading to the dissolution of crystals. In contrast, when the suspension was not stirred, approximately 12% of the crystals were found in the suspension, Figure 10a. In this set of experiments, a higher yield of crystals was achieved when the suspension was not mixed. Moreover, when comparing the values of dissociated cations and crystals on the membrane, the ratio of these values is almost the same. Since the goal of the process is to dissolve only the bottom layer of crystals and release them into the suspension, it is evident that intensive mixing in the reactor is unsuitable. This is because it facilitates the transfer of protons, which subsequently neutralize all available products, including the portion that has already entered the suspension.

#### 3.3.2. Change in the Acid Flow Rate Within the Fiber

Based on previous results, the suspension has not been stirred in this set of experiments and investigated the effect of the volumetric flow rate of the acid within the fibers on the yield of crystals in the suspension. The fiber utilized is relatively lengthy, and the driving force for proton transport between the module’s inlet and outlet can be considerable. This variation in driving force can notably influence the efficiency of proton transfer through the membrane, thereby affecting the overall effectiveness of crystal detachment. An increase in the flow rate of HCl leads to a faster delivery of H^+^ ions. Thus, a higher volumetric flow rate of the acid increases the potential for disrupting the lower layer of crystals and promoting their successful detachment from the fibers of the module.

At the end of the third stage, in the first two experiments where the volumetric flow rate was 14 mL/min and 28 mL/min, the crystal yields were almost identical, at 7.20% and 7.60%, respectively, Figure 10b. Ultimately, the yield of crystals in suspension increased with an increase in acid flow rate. The highest yield was achieved 9.56%, if the volumetric flow was 42 mL/min. Moreover, if the percentage values of dissociated magnesium cations are compared, the differences between the values are not as pronounced as in other sets of experiments.

#### 3.3.3. Variation in the Stirring Rate of the Suspension

**Figure 11:**
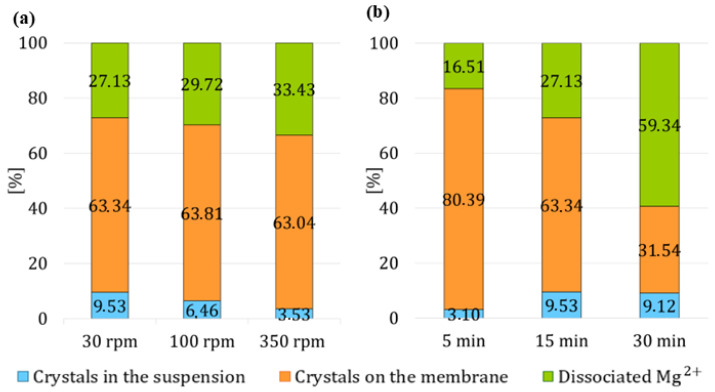
The percentage of reacted and dissociated Mg^2+^ ions in the third experimental stage with varying (**a**) HCl volume flow, (**b**) time duration of HCl circulation.

On the basis of previous experiments, the effect of the intensity of the stirring speed of the suspension was investigated. Although intensive stirring of the suspension negatively affects the proportion of crystals present in the suspension, it also facilitates the transfer of H^+^ protons and the detachment of crystals from the membrane surface into the suspension. For this reason, the effect of stirring was studied at three different speeds—30 rpm, 100 rpm, and 350 rpm. Differences only became apparent in the third stage, when the suspension was mixed more intensively, with the final result being shown in Figure 11a. If the suspension was mixed at 30 rpm during the second stage, the highest yield of crystals in suspension was achieved after the third stage in this set of experiments at 9.53%. At this stirring speed in the second stage, the crystals were most probably not detached into the suspension. Instead, only the bottom layer of crystals near the vicinity of the membrane were dissolved, and when mixing was intensified in the third stage, the top layer of crystals was released into the suspension, with only a small percentage of crystals dissolving. In the experiments where the suspension was stirred at higher speeds, the percentage of dissociated magnesium cations in suspension increased to 33.43%. Compared 33.43% and 27.13% of dissociated cations from the experiments with suspension mixing at 350 rpm and the experiment with suspension mixing at 30 rpm, representing an increase in dissociated magnesium cations of more than 6%, which indicates that dissolution of crystals in the suspension occurs with increasing stirring speed of the suspension.

The results from this set of experiments confirmed that more intensive stirring in the second stage facilitates H^+^ transport through the membrane leading to dissolution of crystallized magnesium hydroxide throughout the whole solution.

#### 3.3.4. Variable Time Duration of HCl Circulation

The results from experiments with varying time duration of the second stage show that circulating HCl for 5 min was not sufficient to detach an adequate amount of crystals into the suspension, when only 3.10% of crystals were in the, see Figure 11b. suspension. Conversely, circulating HCl for 30 min increased the portion of crystals in suspension to 9.12%, but this came at the expense of dissociated magnesium cations, whose percentage in the suspension increased more than fivefold to 59.34% compared to 16.51% in the experiment with 5 min second stage. The results suggest that if crystal detachment into the suspension occurred, the acid feeding duration was excessively long, leading to a dissolution of the crystals in the suspension. As a result, crystal dissolution occurred not only on the membrane surface but also within the suspension. If HCl was circulated for 15 min, the highest yield of crystals in suspension in this set was achieved at 9.53%, while the percentage of dissociated cations increased to 27.13% which is 8% higher than in the experiment with a 5 min HCl circulation (16.51%). From this, it can be concluded that crystals were primarily detached from the membrane into the suspension. From this set of experiments, it would be more advantageous to conduct the second stage for a duration of 15 min, because the suspension after the experiment contained twice as few dissociated magnesium cations compared to the experiment with a 30 min second stage and three times more crystals in the suspension compared to the experiment with a 5 min second stage.

### 3.4. Evaluation of Modifications Applied in the 3rd Stage

Conditions for the second stage were determined based on previous sets of experiments. The most suitable conditions were the least intense stirring speeds of the suspension at 30 rpm for 15 min.

#### 3.4.1. Impact of Stirring Speeds of the Reactor

**Figure 12:**
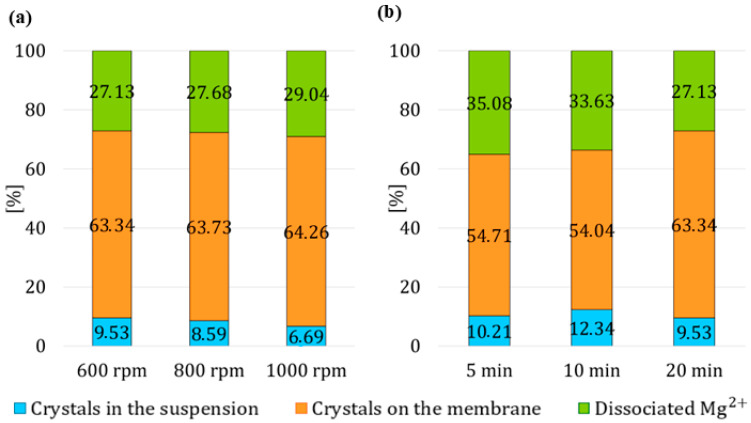
The percentage of reacted and dissociated Mg^2+^ ions in the third experimental stage with varying (**a**) stirring speed, (**b**) time duration of the third stage.

At stirring speeds of 600 rpm, 9.53% of crystals were present in the suspension, at 800 rpm, their portion decreased to 8.59%, and at 1000 rpm, the percentage of crystals in the suspension dropped to 6.69%, as shown in Figure 12a. The portion of the crystals on the membrane remain relatively stable across all stirring speeds tested, ranging between 63.34% and 64.26%. High mixing speeds could not only cause the detachment of crystals in the suspension but also their fragmentation into smaller crystals. Additionally, the water introduced into the module could lead to the additional expulsion of a small amount of H^+^ ions present in the membrane pores from previous stage, which then caused the dissolution of the crystals, as indicated by the concentration of dissociated magnesium cations, which slightly increased with stirring speed—from 27.13% to 29.04%. The results of this set show that intensified mixing of the suspension during the third stage caused a decrease in the yield of crystals in the suspension.

#### 3.4.2. Effect of Time Duration of Circulation During the Third Stage

The data reveals that the proportion of crystals in the suspension increases slightly from 10.21% at 5 min to 12.34% at 10 min, then decreases to 9.53% at 20 min. This trend suggests that moderate circulation time promotes the detachment of crystals into the suspension. The portion of crystals on the membrane (orange segments in Figure 12b remains relatively stable between 5 and 10 min, approximately 54%, but increases significantly to 63.34% after 20 min of water circulation. Simultaneously, the percentage of dissociated Mg^2+^ ions decreases from 35.08% at 5 min to 27.13% at 20 min.

## 4. Conclusions

The presented study investigated methods to enhance the efficiency of magnesium hydroxide reactive membrane crystallization using a hollow fiber anion-exchange membrane, with the aim of increasing the yield of crystals in the suspension, thereby minimizing membrane fouling and improving product recovery. The membrane crystallization process demonstrated potential for magnesium recovery, achieving a conversion consistently over 85%. However, the majority of produced crystals remained attached on the membrane surface. At the end of the membrane crystallization stage, the suspension contained, on average, only 1% magnesium hydroxide crystals, indicating significant product scaling. To address this, the experiments were designed as batch processes, each consisting of three consecutive stages. The first stage involved 5 h of membrane crystallization under controlled conditions. This was followed by a second stage in which hydrochloric acid was circulated through the membrane to dissolve part of the adhered crystal layer and facilitate detachment. Finally, a third stage involved the circulation of demineralized water to assess further removal and redistribution of the crystals into the suspension. Throughout all three stages, operational parameters such as acid circulation duration and suspension mixing intensity were varied to evaluate their influence on the yield of suspended crystals. This approach allowed for a systematic investigation into in situ methods for scale removal and product recovery, offering practical insight into optimizing membrane crystallization for applications in resource recovery and zero-liquid discharge systems.

The results demonstrated that the timing, duration and intensity of process modifications in each stage significantly influenced the distribution of magnesium species between the membrane, suspension, and its dissociated forms. Introducing HCl in the later stages of the first stage resulted in increased detachment of crystals from the membrane without excessively increasing the concentration of dissociated magnesium ions. In the second stage, moderate conditions for acid circulation of 15 min and low stirring intensity resulted in the highest crystal yield in suspension (9.53%) while minimizing dissolution. Excessively long acid exposure or vigorous mixing promoted undesired dissolution of both membrane-bound and suspended crystals, resulting in more than a twofold increase in the concentration of dissociated magnesium. Similarly, in the third stage, a moderate water circulation time of 10 min at a controlled stirring intensity of 600 rpm achieved the highest overall magnesium hydroxide yield in the suspension: 12.34%. However, a compromise is desired between maximizing suspended crystal recovery and minimizing dissociated magnesium concentration in the solution.

The study confirms that targeted manipulation of process parameters can lead to greater product recovery in the bulk stage. While the demonstrated methods resulted in an approximate 10% increase in the proportion of suspended crystals, it is important to note that the experiments were conducted in batch mode at small scale, using magnesium concentrations typical of desalination brines. As a result, the absolute yield of product was relatively low. However, the findings validate the potential of in situ scale removal strategies and provide a foundation for optimizing reactive membrane crystallization systems—not only for magnesium hydroxide but potentially for other valuable salts in high-salinity streams.

Future work will focus on scaling up the process to continuous operation, using multi-fiber membrane modules. The flux per unit membrane area will be the same. The challenge will be to ensure that all fibers in the module are effectively utilized. In this envisioned setup, a dedicated membrane crystallization stage would be followed by a crystal harvesting stage, enabling sustained operation and higher overall product recovery. Additional strategies, such as integrating gas bubbling into the suspension, will also be explored to further enhance crystal detachment and improve system efficiency at scale.

## Figures and Tables

**Table 1:** Cation composition of exhausted brines.

Source	Concentration of Main Cations [g/l]			
	Na^+^	K^+^	Ca^2+^	Mg^2+^
Sea water [3]	10.8	0.39	0.41	1.29
Mine brine [5]	36.84	0.65	2.52	2.84
Trapani saltworks brine [4]	48.30	8.62	0.46	36.06
Ionic exchange resins brine [6]	26.15	-	24.12	3.08

**Table 2:** Anion-exchange membrane properties and module characteristics.

Item	Value	Unit
Outer diameter	2	mm
Inner diameter	1.5	mm
Membrane thickness	0.25	mm
Total length	150	cm
Ion-exchange capacity	2	meq/g
Active surface of the dry module	94.25	cm^2^
Active surface of the swollen module	100.85	cm^2^

**Table 3:** Established operational parameters for membrane crystallization.

Item	Value	Unit
Concentration of MgCl_2_	9.52	g/L
Concentration of NaOH	20	G/L
Volumetric flow rate	14	ml/min
Agitator speed in the reactor	600	rpm
Agitator speed in the reservoir	100	rpm
Duration of the experiments	300	min

## Data Availability

The data presented in this study are available on request from the corresponding author.

## Abbreviations

The following abbreviations are used in this manuscript:

MCr	Membrane crystallization
RO	Reverse osmosis
SEM	Scanning electron microscope
